# The use of foundational ontologies in biomedical research

**DOI:** 10.1186/s13326-023-00300-z

**Published:** 2023-12-11

**Authors:** César H. Bernabé, Núria Queralt-Rosinach, Vítor E. Silva Souza, Luiz Olavo Bonino da Silva Santos, Barend Mons, Annika Jacobsen, Marco Roos

**Affiliations:** 1 Department of Human Genetics, Leiden University Medical Center, Leiden, The Netherlands; 2 University of Twente, Enschede, The Netherlands; 3 Federal University of Espírito Santo, Vitória, Brazil

**Keywords:** Systematic literature mapping, Foundational ontologies, FAIR, Biomedical ontologies

## Abstract

**Background:**

The FAIR principles recommend the use of controlled vocabularies, such as ontologies, to define data and metadata concepts. Ontologies are currently modelled following different approaches, sometimes describing conflicting definitions of the same concepts, which can affect interoperability. To cope with that, prior literature suggests organising ontologies in levels, where domain specific (low-level) ontologies are grounded in domain independent high-level ontologies (i.e., foundational ontologies). In this level-based organisation, foundational ontologies work as translators of intended meaning, thus improving interoperability. Despite their considerable acceptance in biomedical research, there are very few studies testing foundational ontologies. This paper describes a systematic literature mapping that was conducted to understand how foundational ontologies are used in biomedical research and to find empirical evidence supporting their claimed (dis)advantages.

**Results:**

From a set of 79 selected papers, we identified that foundational ontologies are used for several purposes: ontology construction, repair, mapping, and ontology-based data analysis. Foundational ontologies are claimed to improve interoperability, enhance reasoning, speed up ontology development and facilitate maintainability. The complexity of using foundational ontologies is the most commonly cited downside. Despite being used for several purposes, there were hardly any experiments (1 paper) testing the claims for or against the use of foundational ontologies. In the subset of 49 papers that describe the development of an ontology, it was observed a low adherence to ontology construction (16 papers) and ontology evaluation formal methods (4 papers).

**Conclusion:**

Our findings have two main implications. First, the lack of empirical evidence about the use of foundational ontologies indicates a need for evaluating the use of such artefacts in biomedical research. Second, the low adherence to formal methods illustrates how the field could benefit from a more systematic approach when dealing with the development and evaluation of ontologies. The understanding of how foundational ontologies are used in the biomedical field can drive future research towards the improvement of ontologies and, consequently, data FAIRness. The adoption of formal methods can impact the quality and sustainability of ontologies, and reusing these methods from other fields is encouraged.

**Supplementary Information:**

The online version contains supplementary material available at 10.1186/s13326-023-00300-z.

## Background

Ontologies have long been used in biomedical research and applications [1]. For instance, these artefacts play an important role in improving the semantics and machine-actionability of Findable, Accessible, Interoperable and Reusable (FAIR) data and resources [2, 3]. Foundational ontologies are high-level, domain-independent ontologies constructed to provide basic categories and relations to concepts in domain-specific ontologies [4]. Theoretically, foundational ontologies are claimed to enhance the quality of domain specific ontologies and facilitate the interoperability among ontologies grounded on the same foundational one [3–6]. However, it is difficult to find empirical evidence testing these claims.

The biomedical field has been developing and reusing tools to deal with the increasing growth in the volume of research data, which is impossible to analyse by human agents alone. As a result, several approaches have been proposed to make data and metadata (i.e., description of data) machine-readable and -actionable, such as to enable computers to understand and automatically process them (e.g., [2, 7]). To that end, the FAIR principles [2] focus on enabling efficient data analysis across multiple resources with minimal human intervention. The realisation of FAIR principles is intrinsically dependent on ontologies since they are used to describe, for instance, catalogues of resources (Findability), machine-readable access conditions (Accessibility), data and metadata (Interoperability and Reusability), and reuse conditions (Reusability) [8, 9].

In several fields, ontologies are used to model, represent, share and process knowledge about a domain. Ontologies became popular among bioinformaticians with the development of the Gene Ontology (GO) in 1998 [10, 11]. The success of GO has led many other groups to develop their own ontologies, which triggered initiatives such as the Open Biological and Biomedical Ontology (OBO) Foundry to coordinate ontology development efforts [11, 12]. To the current date, there are more than 250 active ontologies registered on the OBO Foundry Portal. Other ontology repositories also list an increasing amount of bio-ontologies. For instance, the NCBO Bioportal [13] catalogue currently contains more than a thousand ontologies.^1^

In general, the use of ontologies in the biomedical field faces several types of challenges. Some authors highlight the inherent diversity of the biomedical domain as one such challenge [14, 15]. This diversity can be perceived when capturing the domain’s different levels of organisation, distinct types of entities, processes and relationships held by each entity. To illustrate, consider the multifaceted nature of proteins, which encompass sequences, functions, location, structure, interactions, related diseases, and so on [14]. This challenge is compounded by the constantly evolving nature of biomedical knowledge, which requires ontologies to continuously adapt as the field changes [1, 14, 15]. Additionally, ontologies play a significant social function by representing the collective knowledge and commitments of the communities that develop and use them [1, 14, 15]. As a result, there is a need for maintaining ontology quality, and fostering community awareness and acceptance of ontologies among all involved stakeholders (e.g., researchers, clinicians, developers, and end-users).

Furthermore, with the growing popularity of ontologies, it is possible to find different ones describing the same or a very similar domain scope. These overlapping ontologies can present conflicting definitions of the same concept, which impacts interoperability [16, 17]. To cope with that, prior literature suggests the organisation of ontologies in levels, where domain-specific (low-level) ontologies are grounded on domain-independent (high-level) ones, also known as foundational ontologies [17–19]. Most foundational ontologies reuse theories from cognitive science, philosophy, logic (i.e., description and first order logic [20]) and linguistics [4] to make clear philosophical distinctions about basic entities of the world [21, 22]. Arguably, these theoretic models can be used to articulate different conceptualisations across domains, and so, to enable interoperability [3]. Foundational ontologies are adopted by several research fields, including biomedical research [23]. Notably, the OBO Foundry defines a set of ontology development best practices that, for instance, proposes that each ontology should be built reusing a foundational ontology (more specifically, the Basic Formal Ontology (BFO) [24]).

The use of foundational ontologies can play an important role in the foreseen world of machine-actionable FAIR data, and also support bio-ontologists in dealing with the aforementioned challenges. However, we argue that this role must be well understood, and its expectations should be supported by empirical evidence. To address such need, this paper describes a systematic study of the literature to understand how foundational ontologies are used in biomedical research and its applications (including related and sub-fields such as bioinformatics). We seek to find empirical evidence supporting the claimed (dis)advantages of using foundational ontologies. Due to an apparent lack of adherence to formal methods in the development and evaluation of ontologies [25], we also explore how biomedical ontologies (developed using foundational ones) are developed and evaluated. Our approach is based on a Systematic Literature Mapping (SLM), which is a method to analyse the state-of-the-art on a particular topic [26].

### Ontologies and ontological levels

Gruber [27] defines ontology as an “explicit specification of a conceptualization”. This definition is extended by Studer, Benjamins & Fensel [28], who define ontologies as a “formal, explicit specification of a shared conceptualization”. ‘Formal’ means that the conceptual model is logically defined so it supports algorithmic reasoning. ‘Explicit’ refers to concepts being defined with unambiguous descriptions. Finally, ‘shared conceptualization’ refers to the consensual definition of domain concepts within the community of expected users.

Usually, ontologies are organised in the application, domain, core and foundational levels [18]. Application ontologies are built to address a specific use case, usually constrained to a particular activity (e.g., orchestrate a machine learning workflow). Domain ontologies describe concepts related to a domain of discourse (e.g., rare diseases). Core ontologies provide an upper-level structural definition of a field that spans across different domains (e.g., biomedicine). Foundational ontologies define high-level (general) and domain-independent concepts (e.g., *process*, *quality*, *object*) that are articulated to define lower-level (more specific) ones. For example, the concept of *mitotic cell cycle* in GO can reuse the basic properties of *process* from BFO, thus inheriting its properties of having temporally proper parts and dependence on some material entities [24].

**Fig. 1:**
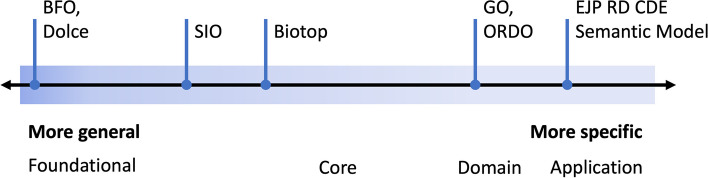
de Almeida Falbo et al.’s view of ontological levels as a continuum. Ontologies that define more general concepts of the world (e.g., foundational ontologies) are placed more to the left, while domain specific ones are placed more to the right. In the examples, BFO is defined in the leftmost side, while Biotop is depicted as more specific than BFO, but still more general than domain ones, such as ORDO. The positioning of SIO, Biotop, GO, ORDO and the EJP RD CDE Semantic Model are examples suggested by the authors of this paper

Some authors suggest that the ontological level should be seen as a continuous scale [19]. Figure 1 exemplifies the view proposed by de Almeida Falbo et al. [19]. In this scale, the boundaries between foundational and core, and between core and domain/application ontologies are not always clearly defined. For instance, BFO is always considered a foundational ontology, positioned on the leftmost side of the continuum (exact classification). On the other hand, the Semanticscience Integrated Ontology (SIO) [29] and Biotop [30] are positioned between foundational and core ontology (our suggestion), since they define both domain-independent but also biomedical related concepts. The Descriptive Ontology for Linguistic and Cognitive Engineering (DOLCE) [31], the General Formal Ontology (GFO) [32], the Suggested Upper Merged Ontology (SUMO) [33] and the Yet Another More Advanced Top-level Ontology (YAMATO) [34] are also examples of foundational ontologies (leftmost side of the scale, exact classification). The Orphanet Rare Disease Ontology (ORDO) [35] and the EJP RD CDE Semantic Model [36] can be seen as examples of domain and application ontologies (our suggestion), respectively. In the literature, foundational ontologies are also termed as “top” or “upper level” ontologies, while core ontologies can be named “domain upper ontologies” or “middle level ontologies”.

## The systematic literature mapping

**Fig. 2:**
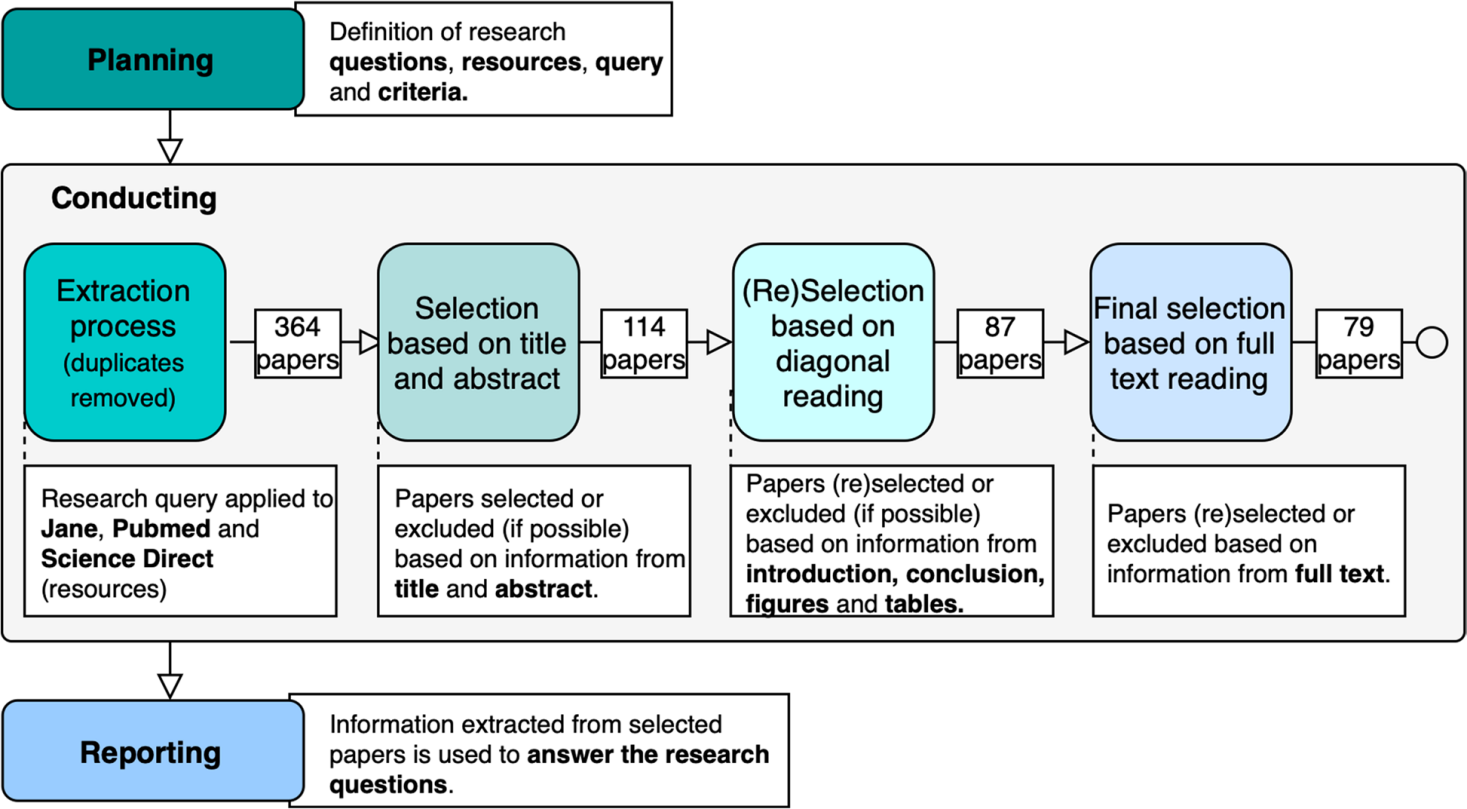
Steps of the SLM method. The first step - planning - consists of defining the research questions, sources, queries and selection criteria. During conduction, the queries are applied to research sources, and the extracted papers are deduplicated and selected accordingly to different excerpts of information. In the last step, the selected papers are used to answer the research questions defined in the first step

The SLM research method used in this literature study is defined by Kitchenham [26] as a secondary study designed to answer broad questions about a research area. Planning, Conducting and Reporting are the main steps of the SLM process and are further divided into more specific tasks (Fig. 2). In the planning step, the research questions, research sources, research query and selection criteria are defined. In the conducting step, papers are extracted from considered sources, deduplicated and selected according to inclusion and exclusion rules. In the reporting step, the results from the SLM are compiled and discussed in the form of answers to research questions.

**Planning.** We defined five research questions (Table 1) in the first task of the planning step. First, we wanted to know “How are foundational ontologies used in biomedical research?” (*RQ1*). Secondly, we wanted to investigate the reason why foundational ontologies are or are not used, and hence we asked two questions: “What are the claimed advantages of using foundational ontologies in biomedical research?” (*RQ2*) and “What are the claimed drawbacks of using foundational ontologies in biomedical research?” (*RQ3*). Third, since the answers to *RQ2* and *RQ3* are based on perceptions by the extracted papers’ authors, we wanted to find scientific support for the answers to the questions by asking “What is the empirical evidence for the advantages and drawbacks of using foundational ontologies in biomedical research?” (*RQ4*). Finally, our second observation could be answered by asking “From the total number of papers that describe the development of a biomedical ontology, how many use existing formal development and evaluation methods?” (*RQ5*).

**Table 1:** Research questions raised in the planning step of the SLM process. The first column identifies the research question, while the second column describes the question itself. The research questions are based on the literature

ID	Research question
**RQ1**	How are foundational ontologies used in biomedical research?
**RQ2**	What are the claimed advantages of using foundational ontologies in biomedical research?
**RQ3**	What are the claimed drawbacks of using foundational ontologies in biomedical research?
**RQ4**	What is the empirical evidence for the advantages and drawbacks of using foundational ontologies in biomedical research?
**RQ5**	From the total number of papers that describe the development of a biomedical ontology, how many use existing formal ontology development and evaluation methods?

Based on our research questions, we selected papers that discuss or use a foundational ontology in biomedical research related domains (Inclusion Criterion - **IC**). We excluded papers that did not mention a foundational ontology at all or were not related to biomedical research (Exclusion Criterion 1 - **EC1**). For the sake of reproducibility of this study, we also excluded papers not written in English (**EC2**). Table 2 summarises the inclusion and exclusion criteria.

**Table 2:** Inclusion and Exclusion criteria defined in the planning step of the SLM method

**Inclusion criteria**	
**IC**	The paper discusses or uses a foundational ontology in biomedical research.
**Exclusion criteria**	
**EC1**	The paper is not related to biomedical research or no foundational ontology was mentioned.
**EC2**	The paper is not written in English.

Sources were selected considering the biological and computational aspects of biomedical research. We included one biosemantics focused source (**Jane** [37]), one biomedical (**Pubmed** [38]) and a third that also covers areas from computer science (**Science Direct** [39]). Finally, the search strategy was driven by the fact that different terms are used to describe foundational ontologies, as mentioned in the previous section. Thus, we included the “top level” and “upper level” synonyms in the search string, which is generically described in Table 3. Some small adjustments had to be done to fit the research string to the sources. For instance, it was not necessary to use the second part of the search string (the one after “AND”) when extracting papers from Jane, since it is already constrained to the biomedical research field. The specific search strings, the search results (and the date each one was performed), are available in the supplementary material.

**Table 3:** Search string defined in the planning step of the SLM. The search string is used to extract papers from the defined sources (Jane, Pubmed and Science Direct)

Search String	
(“foundational ontology” OR “top-level ontology” OR “top level ontology” OR “upper-level ontology” OR “upper level ontology” OR “upper ontology”) AND (“biology” OR “biomedical” OR “biomedicine” OR “biological”)	

**Conducting.** In the extraction process, the search string was used in the mentioned sources and applied to the paper’s full-text search. The search result was downloaded from each source, merged, deduplicated and selected according to the criteria defined. As described in Fig. 2, the selection process is performed in three steps, where papers are first selected based on information from the title and abstract. Then, the results from the first step are reanalysed, now by performing diagonal reading (introduction and conclusion sections, figures and tables). In the third step, papers from step two are definitely selected/excluded based on full-text reading.

**Reporting.** In this final step, we compiled and reported the analysis conducted on the resulting selection of papers. During the final iteration of the selection process, the information from the papers was manually annotated and combined into mind maps that grew iteratively with each reading of a new paper. The mind maps and the information about the extraction process (which criterion was applied to each paper in each phase) are available as supplementary material.

## Results

As illustrated in Fig. 2, the first step of the extraction process resulted in 426 records, which were then deduplicated, resulting in 364 papers. The final step of the extraction process resulted in 79 papers, which comprehend works published in the years from 2004 to 2021. Overall, the number of publications per year is considerably stable, with peaks of nine papers dating from 2011, 2012 and 2015. DOLCE was the most used ontology in the years from 2004 to 2008, while BFO gained prominence from 2010 onwards. Additionally, BFO is the most used foundational ontology among the set of selected papers (42 papers), followed by Biotop (20), DOLCE (17), GFO (8), SIO (3), SUMO (3) and YAMATO (1). It is important to notice that some works used more than one foundational ontology in combination. We consider that a foundational ontology is “used” when it is applied to one of the activities described in the answer to *RQ1*.

From the set of selected papers, 57 of them developed or applied ontologies to specific fields of biomedical research, such as diseases (e.g., [40]), epidemiology (e.g. [41]), and genetics (e.g., [42]). The remainder of them (22) discussed or reviewed philosophical or logical aspects related to foundational ontologies, such as part-whole relations (e.g., [43]), granularity (e.g., [44, 45]) and dispositions (e.g., [46]). Following the SLM steps, we analysed the set of selected papers to answer the five research questions previously defined.

### Synthesised responses to research questions

The answers presented next synthesise our results. Detailed information can be found in the supplementary material, and a summary of the answers that follow is depicted in Table 4. Answers to *RQ1* (“How are foundational ontologies used in biomedical research?”) can be classified into four categories:

- **Ontology development:** in most cases, foundational ontologies were used as a starting point for ontology design, providing a set of basic categories for deriving domain concepts (top-down approach). Foundational ontologies were also used in bottom-up (existing domain concepts are anchored in foundational ones), or middle-out (hybrid) approaches. For instance, Jensen et al. [47] used BFO to design the Neurological Disease Ontology (ND) in a hybrid approach. The authors first defined high-level classes and core entities that represent the domain using a top-down method (e.g., ‘disorder’ is_a ‘material entity’, ‘diagnosis’ is_a ‘generically dependent continuant’). Then, primary literature and other clinical knowledge sources were used to identify more specific terms that were then connected to the high-level classes in a bottom-up strategy (e.g., ‘protein aggregate’ is_a ‘disorder’, ‘diagnosis of Alzheimer disease’ is_a ‘diagnosis’). In summary, BFO supported both the characterisation of high-level classes and the categorisation of lower-level ones according to their nature (e.g., ‘independent continuant’ vs ‘dependent continuant’). Foundational ontologies also supported the development of ontology design patterns (e.g., [48, 49]). For example, Schulz et al. [40] used BFO and Biotop to develop a design pattern to support distinguishing the structural, dispositional, and processual aspects of pathologies. The resulting design pattern reused the classes material entity, disposition, and occurrent to articulate the different interpretations of the pathology concept.
- **Ontology analysis and repair:** The ontological categories, relationships, constraints, and axioms defined by foundational ontologies were used to identify and repair inconsistencies in domain ontologies and other informational artefacts (i.e., information systems, databases, information flow processes, or documents), or to perform analysis to identify opportunities for improvement (e.g., clarity, accuracy). For instance, domain ontologies were grounded on foundational ontologies, which allowed for the identification and correction of inconsistencies and improvement of automated inference. To illustrate, Hoehndorf et al. [50] used fragments of BFO, DOLCE and GFO to identify contradictory class definitions across different biomedical ontologies. The authors found inconsistencies when interoperating the term secretion from GO and UBERON (Uber-anatomy Ontology) by applying the process and material object concepts from foundational ontologies. While GO treats secretion as a process, UBERON refers to it as a material object, which makes them incompatible (i.e., disjoint classes) even though they share the same label. Another example includes the work of Pisanelli et al. [51], in which theories from DOLCE were articulated to demonstrate the polysemy of the term “inflammation”. In this case, it was concluded that the “inflammation” term could be refined into physiological function, a characteristic portion of a body part, a clinical condition, and a diagnosis applicable to that condition.
- **Ontology merging and mapping:** Foundational ontologies were used to support merging or development of mappings between different ontologies, usually with the aim of improving interoperability. In this case, foundational ontologies acted as semantic bridges between domain concepts, hence optimising the mapping process. For instance, in the work of Brochhausen et al. [48], the OMIABIS (Ontologised MIABIS) and BO (Biobank Ontology) ontologies, which are both grounded on BFO, were merged using upper-level BFO classes to create the Ontology for Biobanking (OBIB). The upper-level classes helped distinguishing and organising concepts related to, for instance, process (e.g., specimen collection) and material entities (e.g., specimen).
- **Ontology-based data integration and analysis:** domain ontologies developed with a foundational ontology were used to perform data integration and analysis. First, by grounding the data on an ontology, it was possible to identify errors, organise the data and connect it with external sources (e.g., other ontologised databases). Second, by being efficiently curated and by using reasoning, the ontology-grounded data could undergo a more significant data analysis (e.g., using machine learning algorithms), which can identify hidden knowledge [52], or present useful results that support clinical decisions [53]. In many cases, the domain ontologies served as common semantic data models for integrating heterogeneous data spread across different sources. In this context, foundational ontologies were expected to improve the axiomatisation and clarity of domain ontologies and data. For instance, Martinez-Costa & Abad-Navarro [54] mention that the use of Biotop enriched the axiomatisation of the common data model developed in the context of their work, which allowed for an unambiguous integration of domain specific knowledge. Additionally, the authors observe that “taxonomic reasoning allows queries to be performed at different granularity levels.”

Answers to the question related to the claimed advantages of foundational ontologies (*RQ2*: “What are the claimed advantages of using foundational ontologies in biomedical research?”) can be grouped into two main categories: **improvement of data** and **improvement of ontologies**. Advantages related to data are: enhancing data consistency and interoperability (by grounding data into unambiguous and interoperable ontological terms) and improving queriability (so ontologised data can be queried using human-readable terms). This is exemplified in the work undertaken by Masuya et al. [55], which uses YAMATO in a top-level ontology-based implementation of the RIKEN integrated database of mammals, which imports from several public knowledge sources (e.g., Ensembl, MGI). According to the authors, the approach allowed for “a consistent and scalable body of information that is interoperable with the global informational whole based on semantic web technology”. Additionally, they state that “the standardized data formulation provided from top- and middle- level ontologies reduces the labor cost of data management through the reduction of unevenness in the operations of individual databases.”

Regarding the improvement of ontologies, foundational ontologies are claimed to:

- improve the semantic understanding of terms and avoid ambiguity (as in the “inflammation” example above);
- enhance reasoning and prevent errors by, for instance, using the axioms added by foundational ontologies;
- speed up ontology development, through the reuse of top-level categories and other ontologies grounded on the same foundational one;
- improve interoperability, based on the idea that ontologies that use the same foundational ontology are expected to interoperate easier;
- facilitate ontology maintainability by reusing categories from foundational ontologies.

Examples of works that mention the advantages of using ontologies include Burek et al. [56], which notes that the “use of a top-level ontology potentially leads to fewer errors in the curation and creation of domain ontologies, a better understanding of the biological concepts and the means for data and ontology integration.” Along the same lines, Keet [57] writes that “using a foundational ontology with its generic categories of entity types and core relationships across subject domains can facilitate bio-ontology interoperation, it speeds up ontology development.”

A convergence to a small set of similar answers was observed when asking *RQ3* (“What are the claimed drawbacks of using foundational ontologies in biomedical research?”). Some works mentioned the complexity brought up by the use of foundational ontologies as the main drawback. This complexity is perceived in the time spent understanding class descriptions and in the high level of familiarity needed with background philosophical theories. Some papers described the difficulty to evaluate the claimed advantages as a demotivation towards using foundational ontologies. Additionally, the number of papers explicitly mentioning drawbacks (6 papers) is relatively smaller than the number of papers explicitly mentioning advantages of using foundational ontologies (43 papers).

By way of illustration, Some et al. [58] discuss certain advantages and disadvantages of using foundational ontologies. One downside entails a “tendency towards more complexity, especially regarding nested axioms”, in which “simplification steps might be necessary when the ontologies are used in large KGs graphs, the performance of which might be affected by overly complex OWL models.” In terms of evaluation, Boeker et al. [59] points to some difficulties that are also applicable to the use of foundational ontologies: “Due to the complex nature of the ontology artefacts, their evaluation is inherently difficult and manifold”, as defining a good ontology depends “on the objectives of the ontology under scrutiny, its philosophical foundations and the intention of the investigator.”

Unfortunately, just one paper specifically presented an empirical assessment to test the claimed advantages and drawbacks of foundational ontologies (*RQ4*). Boeker et al. [59] conducted a controlled trial to test the hypothesis that “students who received training on top-level ontologies and design patterns perform better than those who only received training in the basic principles of formal ontology engineering.” In the assessment phase, students were asked to solve problems related to different topics, producing a set of ontological models that were compared to a gold standard. However, “the experiment showed no significant effect of the guideline-based training on the performance of ontology developers.” The authors argue that, due to limited methodology, “the study cannot be interpreted as a general failure of a guideline-based approach to ontology development.”

The last question aims to assess the methodological rigour in the process of building and evaluating ontologies (*RQ5:* “From the total number of papers that describe the development of a biomedical ontology, how many use existing formal development and evaluation methods?”). A subset of 49 of the 79 selected papers developed a new domain ontology using foundational ontologies. From these, 39 designed the ontologies using OWL [60], and 10 described the ontologies using other languages such as UML [61] (e.g., [62]) and FOL [63] (e.g., [64]). When analysing the subset of 49 papers, we investigated how they addressed ontology engineering [65] and ontology evaluation [66].

**Ontology engineering.** Six papers stated that ontologies were built following the OBO principles [67]. As shown in Table 5, among the 49 papers that developed ontologies, four used Ontology Development 101 (OD101) [68], two used OntoSpec [69], one used the eXtensible ontology development principles (XOD) [70], one used Good Ontology Design (GoodOD) [71], and one used a combination of methods (OD101 and Methontology [72]). Two papers reused Ontology Design Patterns (ODPs) [73] to develop their own ontology. The other 33 papers did not use any method (ad hoc).

**Table 4:** A summary of the synthesised responses to research questions

ID	Question	Summarised answer
**RQ1**	How are foundational ontologies used in biomedical research?	Foundational ontologies have been used in the development of domain ontologies and design patterns, ontology analysis and repair, ontology merging and mapping, and ontology-based data integration and analysis.
**RQ2**	What are the claimed advantages of using foundational ontologies in biomedical research?	The advantages of using foundational ontologies can be classified into two groups: improvement of data and improvement of core or domain ontologies. The former includes enhancing data consistency, interoperability and queriability. The latter is related to the improvement of semantic understanding of ontological terms, reasoning, inconsistencies prevention, ontology interoperability, maintainability, and a faster development process.
**RQ3**	What are the claimed drawbacks of using foundational ontologies in biomedical research?	The drawbacks of using foundational ontologies are related to their complexity and the difficulty in evaluating their claimed advantages.
**RQ4**	What is the empirical evidence for the advantages and drawbacks of using foundational ontologies in biomedical research?	We identified only one paper that performed an empirical assessment of the use of foundational ontologies in a biomedical research-related setting. The experiment did not reach any conclusion due to limited methodology.
**RQ5**	From the total number of papers that describe the development of a biomedical ontology, how many use existing formal ontology development and evaluation methods?	A subset of 49 papers developed a domain ontology. Among those, 16 used an ontology engineering method from the literature, and 34 performed a certain type of ontology evaluation.

**Table 5:** Description of the ontology engineering methods used in 16 papers among the selected ones. The first column describes the name of the method, while the second mentions the number of papers that followed the method

Ontology engineering method or guideline	Number of papers using the method or guideline
OBO Principles	6
Ontology Development 101 (OD101)	4
Ontology Design Patterns	2
OntoSpec	2
XOD	1
GoodOD	1
Methontology + OD101	1
**Total uses of approaches**	**16**
ad hoc	33

**Ontology evaluation.** As described in Table 6, twenty-one papers evaluated their ontologies by using them in real-world or simulated application scenarios, more specifically: in data integration experiments, to support the development of an information system, in data classification algorithms, in ontology mapping experiments, and in querying and text mining tasks. Three papers used Competency Questions [74] as verification activities. Four papers performed instantiation [65] as a validation step and 3 papers validated the ontologies with domain experts. Two papers evaluated the ontology both through use case scenarios and domain experts. One paper used the *oQual* method [75]. Other studies (15) have not mentioned any kind of ontology evaluation.

**Table 6:** Description of the ontology evaluation approaches used in the studies. The first column describes the approach followed. The second column shows how many papers used the approach

Ontology evaluation and/or validation method	Number of papers using the method
Application to use case	21
Ontology instantiation	4
Competency questions	3
Validation with experts	3
Application + validation with experts	2
*oQual*	1
**Total uses of approaches**	**34**
No mention of evaluation	15

## Discussion

In this study, both the answers and the lack of answers to the research questions can be seen as results. Regarding *RQ1* (“How are foundational ontologies used in biomedical research?”), we found that foundational ontologies are mostly used in activities related to the development, mapping and repair of biomedical ontologies. Some papers also explored the ontology-based data analysis. We also observed that foundational ontologies were used in these tasks to support dealing with the challenges related to the diversity and complexity of the biomedical domain. As mentioned in the results section, several authors used foundational ontologies during ontology development to improve the semantic understanding of terms, to avoid ambiguity, to facilitate ontology maintainability and to improve ontology interoperability.

In general, we hypothesise two possible causes of the perceived complexity of using foundational ontologies (*RQ3*). The first hypothesis is that foundational ontologies are developed with excessive complexity and can be simplified. The second hypothesis, which would refute the first one, is that foundational ontologies are complex by nature, being complex solutions developed to solve complex problems (e.g., the inherent diversity of the biomedical domain).

Additionally, we argue that the answers to *RQ2* and *RQ3* (“What are the claimed advantages/drawbacks of using foundational ontologies in biomedical research?”) need to be examined further before drawing any conclusions, as they may be perceived differently by different people. One concern is related to the expertise of researchers mentioning the (dis)advantages of using foundational ontologies: are they ontology experts or biomedical experts trying to develop an ontology? Do these two kinds of researchers perceive complexity similarly or differently?

Another concern pertains to the influence of various tooling, modelling approaches (i.e., bottom-up, middle-out, top-down), and representation languages (e.g., OWL, FOL) on the ontologists’ perception of foundational ontologies. For instance, we observed that OWL was used to represent a significant number (39 of 49) of ontologies newly developed. Indeed, this is also noted in the work of Flügel et al. [20], who mention that OWL is more popular with developers because of its relatively user-friendly learning curve compared to, for example, FOL. However, due to its limited expressiveness, OWL cannot convey many ontological differences that are studied by foundational ontologists. Therefore, we anticipate that ontology developers engaged with FOL, who typically operate within a more advanced complexity stratum, will likely perceive foundational ontologies as less complex artefacts in contrast to those working within the lower complexity tier of OWL. Nevertheless, despite being significant questions for the research on the use of foundational ontologies, assessing these aspects within a SLM is a challenging task, as it would be difficult to measure the domain complexity and the experience level of all authors of the 79 papers, since this information is usually not available. Future experimentation aimed at assessing the claimed (dis)advantages of using foundational ontologies should consider these hypotheses and aspects.

We see the lack of answers to *RQ4* (“What is the empirical evidence for the advantages and drawbacks of using foundational ontologies in biomedical research?”) as the main finding of this study. We identified only one paper ([59]) that ran an empirical experiment to test the use of foundational ontologies in the development of an ontology in the biomedical domain. It is important to note that the lack of experiments in biomedical literature does not imply that the claimed (dis)advantages of using foundational ontologies are under- or overrated. Actually, it indicates a clear need for evaluating these claims within the biomedical domain and testing the extended benefits for its applications.

Works in other research fields performed evaluations to provide empirical evidence for using foundational ontologies, and their results might be generalised to biomedical research (e.g., [5, 76, 77]). For instance, to test the usefulness of foundational ontologies in ontology engineering, Keet [5] conducted an experiment where participants had to choose between developing a “Computer Ontology” from scratch or reusing DOLCE or BFO. The study concluded that advantages brought up by using foundational ontologies make up for the time spent getting acquainted with them.

Verdonck et al. [77] also experimented the use of foundational ontologies in ontology development. Their work tested the differences between traditional conceptual modelling and ontology-driven conceptual modelling, in which foundational ontologies were used. The authors found out that few differences (e.g., number of ambiguities and inconsistencies) were noticed when participants had to model simple aspects of a domain. However, significant improvements were perceived when participants modelled more complex scenarios. We hypothesise that Verdonck et al.’s finding can explain the results of Boeker et al.’s experiment (from *RQ4*) because the models compared in the latter might not have been of significant complexity.

Finally, in *RQ5* (“From the total number of papers that describe the development of a biomedical ontology, how many use existing formal ontology development and evaluation methods?”) we investigated how ontologies were built and evaluated. Our results show that only 16 of 49 papers used a systematic ontology engineering method or a set of guidelines. Similarly, only 4 of 49 papers used a formal evaluation method (Competency Questions or *oQual*) despite testing the ontology with a general approach (i.e., application or use cases, validation with experts, querying or instantiation).

Although foundational ontologies are claimed to impose a certain level of rigour during ontology development and evaluation, these processes need to be supported by additional techniques [65]. We theorise that using ontology engineering and evaluation methods should be an important concern in the research and development of ontologies, and that evidence is needed to demonstrate their benefit for biomedical applications. These methods guide ontology designers, data stewards and bioinformaticians in defining aspects related to the quality of content and sustainability of ontologies and ontology-based conceptual models (e.g., continuous integration, maintainability, documentation), which consequently impact the long-term realisation of the FAIR principles. In addition, ontology evaluation intends to identify inconsistencies in the developed ontologies, which should improve interoperability. The evaluation using use case scenarios is necessary, and it was done by several papers, but it also needs to be planned and performed with considerable rigour [78] and preferably combined with different approaches. Other research fields, such as the computer science domain, acknowledge that using ontology engineering best practices improves ontology consistency [3]. To exemplify, the research on ontology-based software engineering has been reusing several approaches from its own area of computer science (e.g., agile methods [79] and goal-modelling frameworks [80]) in ontology development. As such, incorporating this formal rigour for biomedical research can have the added value of increasing the FAIRness of ontologies and ontologised data.

Simon et al. [81] also mention that there are understandable reasons for the ad hoc features of many biomedical ontologies (e.g., lack of systematic ontology engineering methods, the non-use a foundational ontology), and we agree with the author’s point of view. Given the urgency to move from paper-based to digital systems, ontologists were forced “to make a series of uninformed decisions about complex ontological issues”, which can be understood in the context of our work as the lack of empirical testing and formal rigour in ontology development. The author also mentions that ontologists have been tempted to seek immediate solutions to particular problems but, to avoid further ad hoc problems, we strongly do not recommend this behaviour in a semantically interoperable digital world. To facilitate the adoption of formal rigour and engineering methods in bio-ontologies development, and to make ontologised data FAIRer, we suggest that both the ontological and biomedical communities work in closer and synergistic collaboration. We may assume that the more the ontology development methods and standards convergence within a community, the better and more interoperable the ontologies will be. Better ontologies will in turn result in better analysis and reuse of FAIR data. The extent to which the application of these methods and standards will translate into benefits for biomedical research will have to be demonstrated.

### Limitations of this review

We recognise that some studies may not have been included in our analysis due to two reasons: (i) a paper may have used a foundational ontology without explicitly mentioning it, or (ii) a paper may have used an ontology that is in a grey area between foundational and core ontologies, and was consequently not properly captured. Additionally, terminological problems in the search string or in the coverage of the databases of the electronic libraries may have led to missing important studies. These can be seen as a trade-off in using a method such as an SLM, since definitions (e.g., whether a foundational ontology was used or not) must be clearly stated so the process can be systematically repeated.

The possible bias in the selection of papers could also have an impact on the results of this SLM. To mitigate this bias, we conducted periodic meetings between co-authors to discuss and validate the preliminary results of our analysis.

Finally, as previously mentioned, certain aspects that could influence the perception of benefits and drawbacks on the use of foundational ontologies in biomedical research (e.g., authors’ experience, domain complexity, design method) were not measured as they could not be assessed from the information available in the papers.

### Recommendations for biomedical applications and research

We expect that the results and discussions presented in this paper will inspire and guide future research and applications of foundational ontologies in the biomedical field. Examples of future endeavours may include tools to support classifying biomedical concepts into foundational ontologies’ classes, domain-specific modelling languages that include theories from foundational ontologies, and efforts to educate people on proper ontology design. In fact, examples of initiatives from other fields are already in place. These include the “BFO classifier” [82], which suggests BFO classes for domain-specific concepts based on the users response to a decision tree questionnaire, and OntoUML [83], which is a modelling language that facilitates conceptual model design while using embedded theories from the Unified Foundational Ontology (UFO) [83]. Additionally, foundational ontologies are already being taught in computer science academic courses (e.g., [84]), and introducing them into biomedical courses would be beneficial.

Finally, other research paths could investigate the benefits of foundational ontologies in areas where they have been little explored, such as in machine learning (ML) and explainable artificial intelligence (XAI) applications. For instance, researchers can investigate whether XAI algorithms perform differently if trained on data that is organised accordingly to a model grounded on a foundational ontology, when compared to the ones trained on unstructured data. Similar investigations are discussed by Amaral, Baião & Guizzardi [6] in their paper about the use of foundational ontologies for data mining. The authors argue that the quality of data mining results is related to the extent that they accurately reflect the real world, and add that the “fundamental ontological distinctions embodied in a foundational ontology can be used to improve the quality of the data mining process, mainly when it includes information from multiple sources that may commit to different theories about a particular concept.”

## Related works

The related works listed in this section have reviewed the literature to investigate the use of foundational ontologies in other fields. Nardi, Almeida & Falbo [85] performed a Systematic Literature Review (SLR) to study the use of foundational ontologies for semantic integration in Enterprise Application Integration (EAI) research. This research area focuses on the development and use of plans, methods, and tools to integrate distinct information systems. They identified that foundational ontologies have been used to solve semantic conflicts between the applications’ concepts, to develop core or domain ontologies, and to integrate different ontologies and databases. The authors also described that most systems and ontologies were developed without any systematic approach (ad hoc).

Elmhadhbi, Karray & Archimède [86] investigated the role of foundational ontologies as a means for the formalisation and integration of heterogeneous resources for information systems. The authors concluded, based on the literature and their own experience, that the “use of upper ontologies improves data quality, reduces development time and especially facilitate large-scale information integration by avoiding ambiguities or inconsistencies to guarantee semantic interoperability of systems.”

Baumgartner & Retschitzegger [87] presented a survey on the use of foundational ontologies for situation awareness, which is a research area that focuses on the decision-making process under complex and dynamic situations. The authors point out three types of uses of foundational ontologies in computational approaches to situation awareness: integration of heterogeneous information, identification of relevant situations in a domain-independent way, and knowledge sharing across domains.

Trojahn et al. [88] performed a survey on the use of foundational ontologies for making domain ontologies interoperable. The work provides an overview of various ontology-matching activities that can benefit from foundational ontologies. They state that the potential of foundational ontologies for clarifying semantics enhances ontology quality, avoids poor ontology design and facilitates interoperability between ontologies. In regard to the challenges, they state that the “problem of matching ontologies gets more complex when involving foundational ontologies”, as it “requires the deep identification of the semantic context, the identification of subsumption relations, and consistency with the formalization.” They conclude that the main challenge in using foundational ontologies relates to the need of specialised knowledge, as using foundational ontologies demands a thorough understanding of their underlying philosophical theories.

Several other works in the literature have also conducted similar reviews and analyses (e.g., [87, 89]). Most of them, including ours, concur on the benefits (e.g., enhance interoperability and semantic clarity) and drawbacks (e.g., complexity) of using foundational ontologies. Likewise, they recognise similar challenges and requirements (e.g., the need for evaluation, systematic approaches and specialised expertise) for advancing the use of ontologies in their field of research. However, none of them have attempted to identify empirical experiments that test the claims of using foundational ontologies. To the best of our knowledge, no studies have reviewed the use of foundational ontologies in the biomedical research field so far.

## Conclusion

This paper described a Systematic Literature Mapping conducted to understand how foundational ontologies are used in biomedical research and to identify the empirical evidence in favour or against claimed advantages. Additionally, we investigated the level of methodological rigour in papers that used foundational ontologies to construct domain ones. Understanding how foundational ontologies are used in biomedical research and applications can better drive future research towards the improvement of ontologies, and consequently the FAIRness of ontologised data. Our findings imply two main conclusions. First, there is a lack of empirical evidence in biomedical literature for or against the use of foundational ontologies. Second, this particular area of biomedical research does not apply ontology development and evaluation more formally and systematically. Consequently, we recommend that research in bio-ontologies addresses the creation or reuse of methods for ontology engineering (considering phases from ontology requirements elicitation to testing and sustainability) and ontology evaluation (encompassing both evaluation techniques and procedures for application-based evaluation) supported by foundational ontologies. Future research could investigate how foundational ontologies are benefiting biomedical applications, how they are used in other fields and what can be reused to improve research in ontologies for biomedicine.

### Supplementary Information

**Additional file 1.**

## Data Availability

The datasets generated and analysed during the current study are available in the Zenodo repository, https://doi.org/10.5281/zenodo.6961846.

## Abbreviations

BFO: Basic Formal Ontology
CDE: Common Data Elements
DOLCE: Descriptive Ontology for Linguistic and Cognitive Engineering
EAI: Enterprise Application Integration
EC: Exclusion Criteria
EJP RD: European Joint Programme on Rare Diseases
FAIR: Findable, Accessible, Interoperable and Reusable
FOL: First Order Logic
GFO: General Formal Ontology
GO: Gene Ontology
IC: Inclusion Criteria
NCBO: National Center for Biomedical Ontology
OBO: Open Biological and Biomedical Ontology
ODP: Ontology Design Pattern
ORDO: Orphanet Rare Disease Ontology
OWL: Web Ontology Language
RQ: Research Question
SIO: Semanticscience Integrated Ontology
SLM: Systematic Literature Mapping
SLR: Systematic Literature Review
SUMO: Suggested Upper Merged Ontology
YAMATO: Yet Another More Advanced Top-level Ontology
UFO: Unified Foundational Ontology
UML: Unified Modeling Language

## Footnotes

1 Information on the OBO Foundry Portal and the NCBO Bioportal checked on July 2023
