# Supplementary figures and images for: The use of foundational ontologies in biomedical research
Source: J Biomed Semantics. 2023 Dec 11;14:21. doi: 10.1186/s13326-023-00300-z (PMC10712036; doi:10.1186/s13326-023-00300-z)

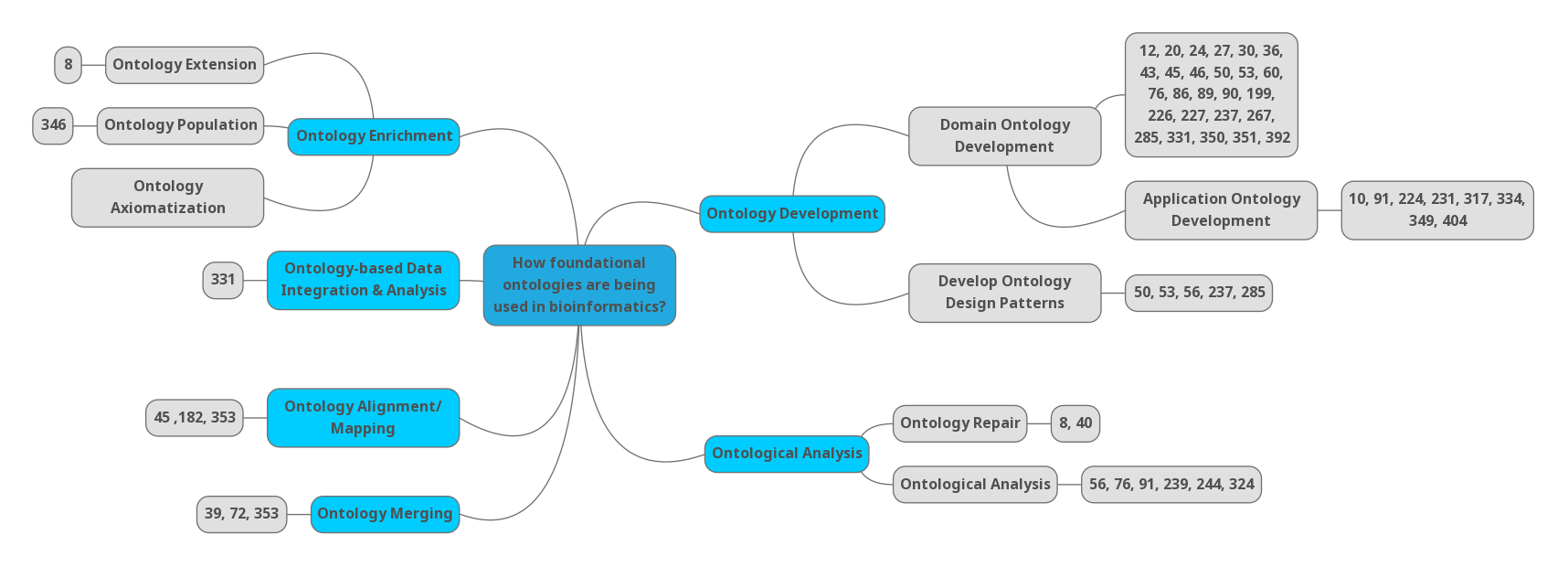

Supplement: Supplementary file 1 — Additional file 1. [file 13326_2023_300_MOESM1_ESM.zip › 13326_2023_300_MOESM1_ESM/Mindmaps/RQ1 - How are foundational ontologies used in bioinformatics.png]

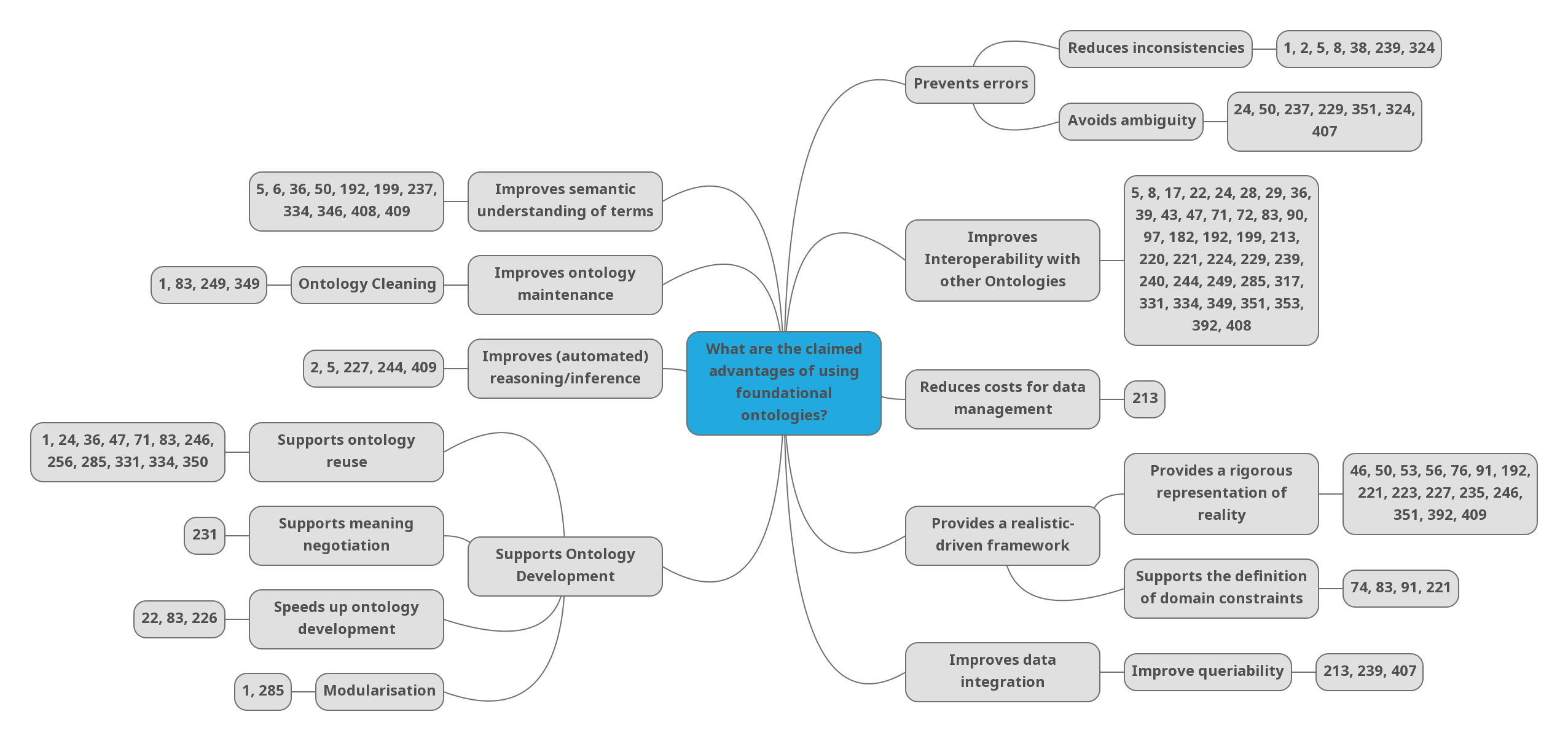

Supplement: Supplementary file 1 — Additional file 1. [file 13326_2023_300_MOESM1_ESM.zip › 13326_2023_300_MOESM1_ESM/Mindmaps/RQ2 - What are the claimed advantages of using foundational ontologies.map.png]

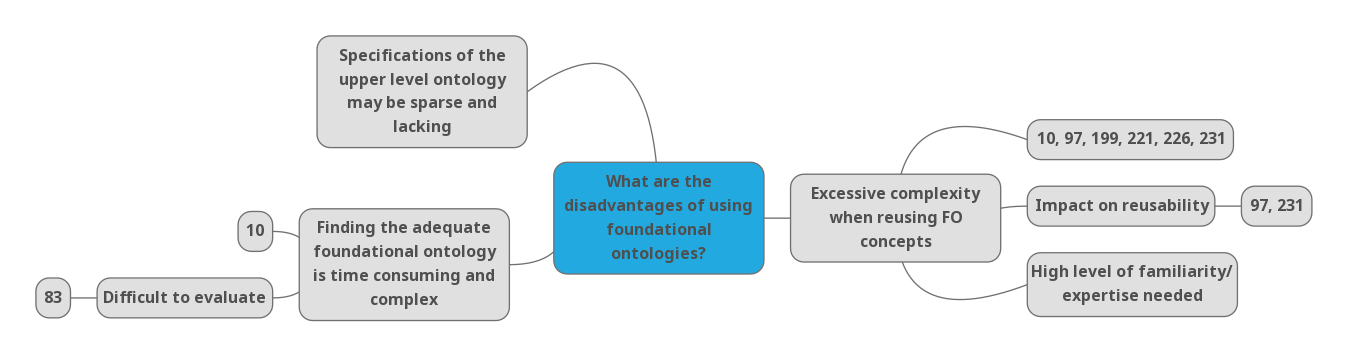

Supplement: Supplementary file 1 — Additional file 1. [file 13326_2023_300_MOESM1_ESM.zip › 13326_2023_300_MOESM1_ESM/Mindmaps/RQ3 - What are the claimed drawbacks of using foundational ontologies.png]
